# Hemostatic Agents in Hepatobiliary and Pancreas Surgery: A Review of the Literature and Critical Evaluation of a Novel Carrier-Bound Fibrin Sealant (TachoSil)

**DOI:** 10.5402/2012/729086

**Published:** 2012-09-13

**Authors:** K. A. Simo, E. M. Hanna, D. K. Imagawa, D. A. Iannitti

**Affiliations:** ^1^Section of Hepatobiliary and Pancreas Surgery, Department of Surgery, Carolinas Medical Center, 1025 Morehead Medical Drive, Suite 300, Charlotte, NC 28204, USA; ^2^Division of Hepatobiliary and Pancreas Surgery and Islet Cell Transplantation, Department of Surgery, University of California, Irvine, Orange, CA 92868, USA

## Abstract

*Background*. Despite progress in surgical techniques applied during hepatobiliary and pancreas (HPB) surgery, bleeding and bile leak remain significant contributors to postoperative mortality and morbidity. Topical hemostatics have been developed and utilized across surgical specialties, but data regarding effectiveness remains inconsistent and sparse in HPB surgery. *Methods*. A comprehensive search for studies and reviews on hemostatics in HPB surgery was performed via an October 2011 query of Medline, EMBASE, and Cochrane Library. In-depth evaluation of a novel carrier-bound fibrin sealant (TachoSil) was also performed. *Results*. The literature review illustrates multiple attempts have been made at developing different topical hemostatics and sealants to aid in surgical procedures. In HPB surgery, efforts have been directed at decreasing bleeding, biliary leakage, and pancreatic fistula. Conflicting scientific evidence exists regarding the effectiveness of these agents. Critical evaluation of the literature demonstrates TachoSil is a valuable tool in achieving hemostasis, and possibly biliostasis and pancreatic fistula prevention. *Conclusion*. While progress has been made in topical hemostatics for HPB surgery, an ideal agent has not yet been identified. TachoSil is promising, but larger randomized, controlled clinical trials are required to more fully evaluate its efficacy in reducing bleeding, biliary leakage, and pancreatic fistulas in HPB surgery.

## 1. Introduction

Improvements in hepatobiliary and pancreas (HPB) surgery over the past 2 decades have resulted in low surgical mortality (<1 to 5%) and morbidity rates (4 to 20%) in high-volume centers [1–3]. However, bleeding (specifically diffuse oozing from the raw resection surface) and bile leakage continue to be problematic in these operations, both intraoperatively, as well as, in the postoperative period. While there are thousands of publications available in various surgical fields regarding the use of hemostats, glues, and sealants; a very small number have focused on HPB surgery [4–6]. Differing requirements and needs for each surgical specialty should lead one to apply caution before inferring equal appropriateness and efficacy of hemostatic (and bilostatic) agents across specialties [7].

A very thorough update and comparison of FDA-approved topical hemostats (capable of clotting blood), sealants (provide a sealing barrier in the presence or absence of blood), and adhesives (bond tissues together) in the “surgical toolbox” was recently performed by Spotnitz and Burks [8, 9]. These authors have previously discussed five performance categories that should be considered in the evaluation of any potential hemostatic agent: safety, efficacy, usability, cost, and approvability [7]. Furthermore, it is stressed that hemostatic agents must be used/applied appropriately so that maximum efficacy can be achieved [8, 9]. They also note that currently, the indications for usage and choice of agents are heterogeneous and for the most part based on the individual surgeon's preference [8, 9]. Likewise, others also note this selection and application is often done without in-depth knowledge of pharmacodynamic characteristics and specific strengths of various agents [5].  Table 1 list all topical hemostatics and sealants currently approved by the FDA as per their website (http://www.fda.gov/) as of December 2011. It is important to note that only a few of the approved hemostatics have a specific indication for HPB surgery. Other FDA approved hemostatics are also frequently used off-label in HPB surgery, as in other subspecialities.

The goal of this paper is to briefly discuss the use of current routine hemostatic agents in HPB surgery and then to perform an in-depth examination of a novel carrier-bound fibrin sealant that permits the simultaneous application of collagen, fibrin, and thrombin (TachoSil, NycomedGmbH, Linz, Austria).

## 2. Methods

**Figure 1:**
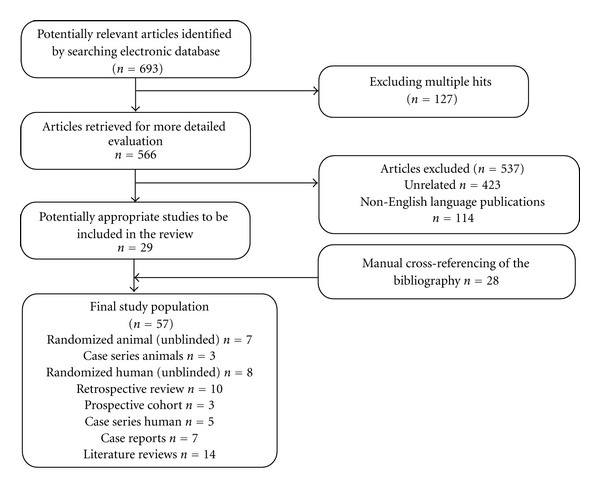
QUORUM flowchart.

Current literature concerning the utilization and effectiveness of topical hemostatic (and bilostatic) agents was reviewed. A comprehensive search for studies and reviews on the use of hemostatic agents in HPB surgery was performed including an October 2011 electronic search of Medline via Pubmed and EMBASE databases and browsing references. Search terms included “hemostat,” “hepatobiliary,” “pancreas,” “liver,” “bile duct,” “fibrin,” “hepatectomy”, and “pancreatectomy”. Also, an in-depth evaluation of a novel carrier-bound fibrin sealant (TachoSil) was carried out with search terms of “TachoSil”, “TachoComb”, and “TachoCombH” in conjunction with “hepatobiliary” and “pancreas”. The article selection process is summarized in Figure 1. Articles were excluded if their focus was not the use of a hemostatic agent, the agent was only mentioned in a description of a procedure, the hemostatic agent was not topical, or hemostasis discussed was not related to hepatobiliary and pancreas surgery. The Cochrane Database of Systematic Reviews was then cross-checked to confirm that no similar reviews have already been undertaken.

## 3. Results

The mainstay of topical hemostatic agents in HPB Surgery have included absorbable gelatin sponges (e.g., Gelfoam, Pfizer, New York, NY), oxidized regenerated cellulose (e.g., Surgicel Ethicon, Inc., Summerville, NJ), gelatin-thrombin matrix (e.g., FloSeal, Baxter Healthcare Corporation, Hayward, CA, USA), collagen and thrombin combinations (e.g., CoStasis Surgical Hemostat, Cohesion Technologies Inc., Palo Alto, CA), synthetic sprayable polymeric matrix (e.g., Coseal, Baxter Healthcare Corporation, Hayward, CA), and/or fibrin glue homemade or manufactured (e.g., TISSEEL, Baxter Healthcare Corporation, Westlake Village, CA, and Crosseal, OMRIX biopharmaceuticals Ltd., Kiryat Ono, Israel, now replaced in the USA by Evicel, Johnson and Johnson, Somerville, NJ). These hemostatics have been used separately and in different combinations with varying success (10 to 60% bleeding complications and 4 to 8% bile leaks reported) [10, 11]. Application of these agents is carried out routinely and without any particular standardized indication. As we progress further into the modern surgical era, the search for an “ideal hemostatic agent” for hepatobiliary and pancreas procedures, although elusive, continues.

Finding this “ideal hemostatic agent” is important, because reduced blood loss and ensuing reduction in blood transfusions has been demonstrated to result in superior surgical outcomes in HPB operations [1]. Furthermore, not only can these agents decrease bleeding, but they may also reduce operative time, improve quality of surgical tissue management and decrease the occurrence of biliary, pancreatic and enteric anastomotic leaks [8, 9]. In patients undergoing a liver resection, decreased liver reserve and cirrhosis need to be taken into account as this can further complicate the achievement of hemostasis following resection.

### 3.1. Current Routine Hemostatic Agents Utilized in Hepatobiliary and Pancreatic Surgeries

Specifically, in regards to hemostasis in HPB surgery, a numerical bleeding score analysis after liver biopsy in a heparinized swine model demonstrated statistically significant hemostatic superiority of porcine gelatin sponge with human thrombin in comparison to porcine gelatin sponge and saline [8, 12]. This superiority was demonstrated in a second study which looked at grade IV-V liver and splenic lacerations in a hemorrhagic shock porcine model where bovine gelatin granules and thrombin (FloSeal) were utilized and found to be effective at achieving hemostasis in all animals [8, 13]. Clot integration was also demonstrated at 48 hours on histological examination [8, 13]. Also, Chapman et al. in a randomized controlled trial of 76 patients undergoing hepatic resection, demonstrated that a mixture of bovine collagen and bovine thrombin is more effective in controlling and stopping diffuse hepatic bleeding than a collagen alone [10, 14].

While value of fibrin as a hemostatic agent was first noted by Bergel in 1909; it was not made commercially available in Europe until 1972, and in 1998 became the first fibrin product approved by the FDA for use in the US [9]. Fibrin sealants remain the only products available in the US with FDA approval for hemostatic, sealant and adhesive bonding indications [9, 15]. In a randomized controlled trial of 121 patients undergoing hepatic resection, 58 patients were randomized to treatment with a 2-component fibrin sealant (Crosseal, Johnson and Johnson, New Brunswick, NJ) and 63 patients to standard topical hemostatic agents such as Gelfoam or Surgicel, used alone or in combination. Fibrin sealant was shown to significantly improve the time to hemostasis in comparison to standard topical hemostatic agents (*P* = 0.003) [16].

Consideration for the usage of topical agents in the prevention of bile leakage in liver surgery is controversial with a plethora of studies supporting both sides of the argument [10]. Likewise, application of tissue sealants and adhesives to seal the transected edge of the pancreas in order to prevent a pancreatic fistula also remains controversial [8].

In a recent prospective randomized study of 300 patients undergoing liver resection, with 150 being treated with fibrin glue after hemostasis was achieved; the primary objective was to determine if fibrin sealant could decrease postoperative blood loss and blood transfusion [4]. Secondary objectives addressed postoperative drainage, incidence of biliary fistula, frequency of reoperation secondary to bleeding or biliary leakage, and frequency of intra-abdominal abscess requiring percutaneous drainage [4]. Tissucol (name under which TISSEEL was marketed in some countries; Baxter-Immuno, Vienna, Austria) in aerosol form was applied to the raw liver surface, followed by application of an absorbable collagen sponge (Johnson & Johnson, Somerville, NJ), with concomitant manual pressure [4]. In comparison to the control group in which no hemostatic agents were utilized, no statistically significant differences were noted for postoperative outcomes, hospital mortality, or overall postoperative morbidity [4]. The authors concluded that application of fibrin sealant on the raw surface of the liver does not seem justified and suggest that discontinuation of routine use of fibrin sealant in these cases. 

### 3.2. TachoSil in Hepatobiliary and Pancreas Surgery

**Figure 2:**
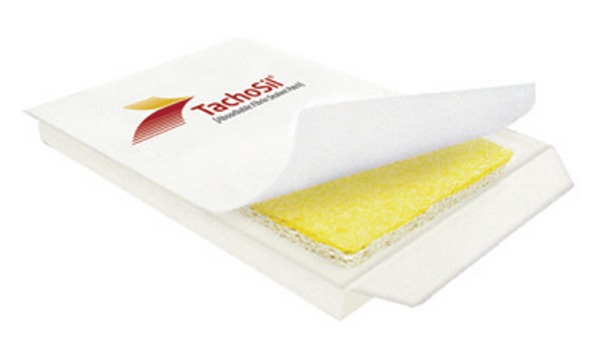
TachoSil packaging.

**Figure 3:**
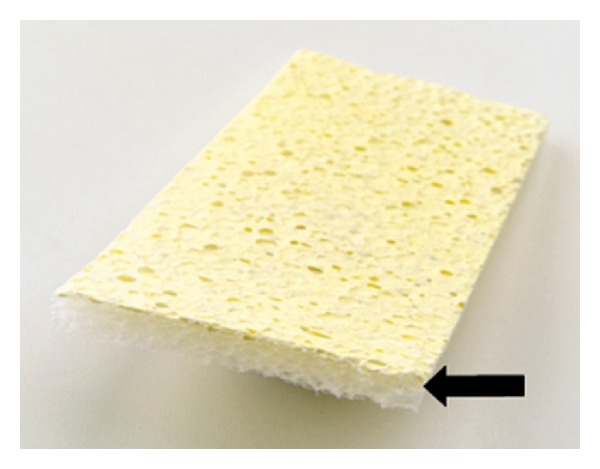
TachoSil Ready-To-Use Surgical Patch. Coating anchored to the indentions as denoted by arrow.

**Figure 4:**
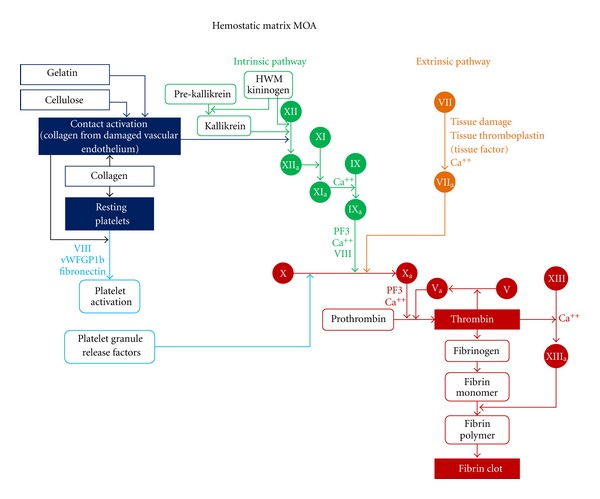
Hemostatic matrix mechanism of action of TachoSil illustrated via the coagulation cascade.

 TachoSil is a ready-to-use fixed combination hemostatic agent consisting of a white honeycomb-like collagen patch coated with the coagulation factors, human fibrinogen, and human thrombin on one side (colored yellow with riboflavin for orientation) (Figures 2 and 3). The patch design takes advantage of the mechanical support of a collagen fleece, as well as the hemostatic and adhesive properties of the coagulation factors I and IIa [2]. Fibrinogen and thrombin are delivered directly to the site of bleeding in order to form a fibrin network effectively gluing the patch to the desired surface (wound, cut liver edge, or anastomosis) [17]. The coagulation cascade is locally activated mimicking the final steps of natural blood clotting to seal tissue [18] (Figure 4). Degradation and reabsorption of the patch and resultant fibrin clot is achieved during the normal healing process [10].

TachoSil represents the most current formulation of a unique carrier-bound fibrin sealant and differs from its precursors, TachoComb and TachoCombH, as earlier components of bovine origin have been eliminated (aprotinin). These precursors were previously approved for use in Europe and Japan (TachoCombH is still in use in some countries but is being phased out and replaced with TachoSil). In the USA, TachoSil was granted approval in 2010 for use as an adjunct to hemostasis for use in cardiovascular surgery when control of bleeding by standard surgical techniques (such as suture, ligature, or cautery) is ineffective or impractical. TachoSil should not be used in the renal pelvis or near the ureter, for skin closures, or neurosurgical procedures [19].

Outside the USA, the current EMA approved indication for TachoSil is for use in adults as supportive treatment in surgery for improvement of hemostasis to promote tissue sealing and for suture support in vascular surgery where standard techniques are insufficient [20]. In addition, TachoSil has also been shown to have multiple other applications including prevention of adhesions and erosions, protection of nerves, and occlusion of structures such as bronchioles, lymph vessels, and bile ducts [21]. Again specifically in this paper, we focus on evaluation of the efficacy and safety of TachoSil and its precursors in HPB surgery.

### 3.3. Abdominal Vasculature

Following extensive HPB surgery, hemostasis following hepatic or portal vein reconstruction can be challenging. This can result from inherent liver disease that is frequently present in this patient population, or in the presence of invasive tumors in the pancreas and duodenum which can be technically difficult to excise predisposing to large volume hemorrhage if injury/laceration occurs. An alternative approach to the traditional repair of vein lacerations using vascular sutures has been studied in the preclinical setting with the use of TachoSil transposed onto a peritoneal patch [22]. In this series, TachoSil was shown to be efficacious in repairing induced inferior vena cava defects in a swine model; the use of a peritoneal patch helps to prevent lumen thrombosis by serving as a barrier from the coagulant portion of the TachoSil sheet. It has also been employed as reinforcement for the sutured anastomosis of the portal vein [23] and has been shown to be useful in the repair of hepatic artery pseudoaneurysm when it develops as a postoperative complication following pancreaticoduodenectomy [24]. Another application of TachoSil for vasculature reconstruction has been described by Shimamoto and colleagues for aortic arch repair [25]. In this study, TachoSil combined pledget stitches which significantly helped to control suture hole bleeding as compared with conventional pledget stitches. This novel application of TachoSil could likely be transferred to intra-abdominal aortic or other large vessel repairs.

### 3.4. Liver Preclinical

The preclinical evaluation of TachoSil has provided evidence for a variety of uses in the field of liver surgery. Early investigations with the TachoSil precursor TachoComb, a collagen fleece patch with fibrinogen, thrombin and aprotinin, have demonstrated initial clinical efficacy for hemostasis following experimentally produced penetrating liver and spleen injury models [26]. Adding to our understanding of hemostasis following liver hemorrhage, TachoSil has also been investigated in an animal model of coagulopathy with blunt liver injury [27]. In this investigation, coagulopathy was achieved by splenectomy and cystotomy followed by hemodilution of 80% blood volume. Blunt liver injury was then induced and the injury treated with either cotton placebo patch or TachoSil fibrinogen/thrombin (FT) patch. All animals treated with the FT patch survived, whereas, all animals in the control group died prior to the end of the observation period. These results demonstrate the efficacy of the TachoSil patch in effectively controlling hemorrhage in the presence of severe coagulopathy.

In a head-to-head trial, TachoSil was compared with a regenerated oxidized cellulose compress (Surgicel, Johnson and Johnson, Somerville, NJ) and a bovine collagen-based compress (Sangustop, B. Braun Aesculap AG, Tuttlingen, Germany) [28]. Liver resection margins were created in a swine model and each of the three compresses applied to different areas of resection margin. Bleeding time and number of compresses required to achieve hemostasis were then measured. The bovine collagen product performed the best in this series with the lowest bleeding time and fewest numbers of compresses required to stop hemorrhage. A second comparative trial of advanced hemostatic dressings evaluated nine different products in a swine model of induced liver venous hemorrhage injury [29]. Four products in this series were excluded from further study secondary to exclusion criteria of no survival or no hemostasis. Of the remaining products evaluated, the American Red Cross fibrinogen and thrombin dressing (currently only available in the USA military) on an absorbable polyglactin mesh were the most favorable in terms of posttreatment blood loss and percentage of animals in which hemostasis was obtained as compared with the TachoComb-S and other hemostatic dressings. In overall, survival during the experimental time period TachoComb-S ranked third.

To examine the effectiveness of TachoSil in sealing of bile ducts, a swine model was utilized in which a medial left liver resection was completed and the cut surface treated with either the fibrin collagen patch or the liquid fibrin sealant (Tissucol Duo 500, Baxter Hyland Immuno, Uden, the Netherlands) [30]. After increasing pressure into the common bile duct, the fibrin collagen patch was found to resist significantly higher intrabiliary pressures prior to bile leakage as compared with the liquid fibrin sealant. Hemostasis in the two groups was equally effective.

### 3.5. Liver Clinical

Use of fibrin-based hemostatic agents and sealants in open liver resection has gained support through numerous publications citing its efficacy in adjunctive hemorrhage control, decrease in postoperative drain fluid output and biliostasis [5, 31–35]. A prospective controlled trial from Briceño and colleagues compared outcomes of 115 patients undergoing major and minor hepatectomies with or without TachoSil as a carrier bound fibrin sealant hemostatic agent [2]. In this series, the TachoSil group was found to be associated with decreased drainage volume (*P* < 0.01), lower volume drain output per day (*P* < 0.01), decreased postoperative blood transfusion rate (*P* = 0.04), shorter mean hospital stay (*P* = 0.03), and fewer moderate to severe postoperative complications (*P* = 0.03). This study's findings are in direct contrast to results published in 2007 in which a comparative cohort study of liver resections performed in 173 patients with TachoComb and 222 patients without TachoComb [36]. No significant differences were seen between groups in rates of postoperative blood transfusion, biliary fistula, or reoperation for postoperative hemorrhage. Currently, a prospective multicentered randomized controlled trial in Austria and Germany is enrolling patients to compare TachoSil, being described as the “gold standard”, with a new microfibrillar collagen hemostat, Sangustop [37]. Termed the ESSCALIVER study, standardization is achieved through resection technique, devices used in surgery, and methods for primary hemostasis. Patients are blinded to group selection and will be followed for three months for postoperative complications and adverse events.

 Additionally, two European trials have demonstrated the hemostatic efficacy of TachoSil as compared with argon beam coagulation (ABC) in liver resection. Frilling and colleagues published results in 2005 following a trial comparing ABC to TachoSil as secondary hemostatic treatment in 121 patients who underwent planned liver resection [38]. In this series, TachoSil performed significantly better in regards to time and to hemostasis (measured from time to application to no visible bleeding evident), 3.9 minutes versus 6.3 minutes, respectively (*P* < 0.01). The investigators also noticed a decrease in drain hemoglobin concentration the second day after surgery in the TachoSil group as compared with the ABC group (*P* = 0.012). No significant difference was seen between groups in regard to adverse events. A follow-up study published by Fischer and colleagues in 2011, was able to replicate some of these findings [39]. In 10 tertiary care centers, 119 patients undergoing liver resection were randomized to receive either ABC or TachoSil. Similar to the Frilling study, the mean time to hemostasis in the TachoSil group was significantly lower than the ABC group (*P* < 0.01). This study did not report however, differences in postoperative drainage volume, drainage fluid, or drainage duration between the two groups. It is important to note that both of these studies were regulatory phase III trials that were aimed at providing data on the hemostatic capability of TachoSil and therefore were not sufficiently powered to determine if any differences exist in postoperative parameters.

Unique considerations exist in the field of liver transplantation as hemostasis, both intraoperative and postoperative can be difficult to achieve and biliary leaks from anastomotic suture lines or cut donor liver surfaces can cause severe postoperative complications. From the pediatric liver transplant literature, the use of TachoSil has been found to be both safe and effective in controlling hemorrhage from split liver donor grafts [40, 41]. Application is directed at all cut-liver surfaces with mild-to-moderate bleeding after primary hemostasis has been achieved. TachoSil has also been found to be effective in decreasing the frequency of bile duct leaks after adult split liver transplantation [42]. From two consecutive cohorts of 16 patients, groups were treated either with the TachoSil or fibrin glue on the cut surface of the donor liver. Bile leaks were found to be significantly fewer in the TachoSil cohort as compared with the fibrin glue cohort (6.25% versus 43.75%, resp., *P* = 0.03).

Application of fibrin sealants and hemostatic agents have gained an increasing presence in the field of laparoscopic liver surgery as new designs for product delivery have been constructed specifically for laparoscopy [33, 43]. TachoSil is approved for laparoscopic surgery in Europe, however its application in laparoscopic liver surgery remains somewhat challenging and depends on individual surgical skill sets for mainly two reasons: (1) the active components can be disrupted from the collagen sheet, particularly when passed through a laparoscopic port and (2) the fibrinogen and thrombin coated sheet, once in contact with blood or body fluids, is activated immediately and thus becomes difficult to manipulate due to its sticky consistency [18]. Innovative techniques for intracorporeal TachoComb delivery have been previously published including a fan-shaped device or small rubber tube to introduce small strips of the hemostatic agent [44, 45]. Carbon and colleagues have also published results of successful hemorrhagic spleen repair using sheets of TachoComb delivered through a special minimally invasive applicator system [46].

At this time, the majority of published reviews of TachoSil in laparoscopic abdominal surgery have been limited to urologic surgery or splenic repair [47–49]. A report from Low and colleagues, describes the use of a liquid fibrin sealant and TachoSil to control a spontaneous splenic capsule rupture during a laparoscopic liver resection for colorectal metastasis [50]. In this case, Pringle maneuver was applied in addition to the hemostatic agents to gain hemostatic control and allow for splenic salvage. Additional studies are needed to further evaluate the role of TachoSil in laparoscopic liver surgery and to compare topical hemostatic agents and their use in laparoscopy.

### 3.6. Pancreas

To date, evidence regarding the use of TachoSil in pancreatic surgery stems largely from retrospective reviews and small case series [51–57]. Anecdotally, this evidence has supported the idea that TachoSil may decrease rates of pancreatic fistula formation secondary to its tissue sealant properties. Investigative reviews however, have not definitively supported these conclusions and conflicting recommendations have resulted. Lorenz and colleagues have reported on a retrospective analysis of 46 distal pancreatic resection comparing stapled versus sutured closure of the pancreatic stump in which TachoComb was applied to approximately 50% of cases in both groups [54]. No significant differences were found in postoperative morbidity or pancreatic fistula rate between groups, but improved outcomes tended to be superior with staple closure, with and without TachoComb. Specifically, even though there were no statistically significant differences, there were fewer leaks (1 versus 7) and none requiring surgical revision in the staple closure group (versus 2 patients with suture closure). No subset analysis was performed of patients who received TachoComb in the suture closure and staple closure groups for determination of pancreatic fistula rate. In another series, patients undergoing open pancreaticoduodenectomy, a Roux-en-Y pancreaticojejunostomy reconstruction was reinforced with TachoSil on the pancreaticojejunal suture line [53]. There were 27 patients in each group; three patients in the non-TachoSil group had a postoperative pancreatic fistula (POPF) while only one in the TachoSil group did. While the results were not statistically significant, investigators have suggested that TachoSil may help prevent POPF.

From the laparoscopic experience, Rosøk and colleagues have reported a 10% pancreatic fistula rate following laparoscopic pancreatic resections including distal pancreatectomy and pancreatic enucleation [56]. Beginning in 2005, the investigators began sealing the resection margin of remaining pancreas with TachoSil; however, they did not report a change in fistula formation following this addition. A second review of laparoscopic distal pancreatic resections in 121 patients by this group found that the addition of the TachoSil patch to the distal pancreatic resection line (also starting in 2005) did not affect occurrence of POPF or the length of hospital stay [55].

 However, supportive evidence has been described for the use of collagen fleece products for hemostatic control in pancreas surgery and in the setting of surgery for acute pancreatitis. Preclinical testing involving animal models have been used to study the hemostatic and sealant abilities of TachoSil under hyperfibrinolytic conditions such as acute pancreatitis [58]. A swine model of acute pancreatitis was induced by retrograde injection of bile into the pancreatic duct with subsequent duct ligation. Hemostatic efficacy was assessed immediately and at 72 hours and was found to be equally effective. Even under conditions of increased intraorgan pressure created by ligation of the splenic vein and administration of adrenaline, hemostasis, and tissue sealing efficacy were not adversely affected by severe hyperfibrinolytic conditions. Furthermore, initial clinical experience with TachoComb, published in 1990, reviewed patients undergoing pancreatic resection for pancreatic carcinoma, necrotizing pancreatitis, and chronic pancreatitis [59]. In this series of 30 patients, collagenic fleece was useful in controlling bleeding from the retroperitoneum and pancreatic bed following resection.

A comprehensive review of fibrin sealants in pancreatic surgery published in 2009 highlights the fact that the body of the current literature does not provide conclusive evidence of the utility of fibrin sealants in pancreatic surgery [15]. At this point, the ability of fibrin sealants to decrease pancreatic fistula rate remains at best, speculative. Randomized large-scale trials are necessary to provide conclusive evidence as to the use of TachoSil and other fibrin sealants in pancreatic surgery. One is currently underway in France and results are expected in mid-to-late 2012.

### 3.7. Current USA TachoSil Trial

Currently, a randomized, open label, parallel group, multicenter trial is underway in the United States to evaluate the use of TachoSil in open liver surgery. In this study, the efficacy and safety of TachoSil will be compared with Surgicel Original for secondary treatment of local bleeding in open hepatic resection surgery. The primary objective is to show that TachoSil is superior to Surgicel Original as a secondary hemostat when conducting hepatic resections. The secondary objective is to evaluate the safety of TachoSil in hepatic resections. The primary endpoint is intraoperative hemostasis 3 minutes after the application of the randomized treatment. Other endpoints considered in this study are the need for additional agents to reach adequate hemostatic control, the number and type of agents applied, and the failure rate with regard to achieving hemostasis. This trial is currently enrolling patients and primary results from this study are anticipated in late 2012.

## 4. Conclusion

 In conclusion, progress continues to be made in topical hemostatic agents for hepatobiliary and pancreas surgery; however, the search for the ideal agent continues. TachoSil is a promising hemostatic agent which is a third generation equine collagen fleece patch, delivering human fibrinogen and human thrombin directly to the site of bleeding for hemostasis and tissue sealing. Its applications in hepatobiliary and pancreas surgery have proven effectiveness in hemostasis and excellence as a tissue sealant. Future randomized controlled trials are needed to determine its ability to control biliary leakage and pancreatic fistula output. Further studies to delineate the role of TachoSil and other fibrin sealants in laparoscopic surgery are also needed to demonstrate improved effectiveness and applicability.

## Figures and Tables

**Table 1:** Topical hemostatics and sealant agents approved by the FDA as of December 2011.

Product	Approval year	Manufacturer	Description	Indication
OMNEX	2010	Ethicon, Inc. a Johnson and Johnson Company, Somerville, NJ	Two cyanoacrylate monomers	For use in vascular reconstructions to achieve adjunctive hemostasis by mechanically sealing areas of leakage.

TachoSil	2010	Nycomed GmbH, Linz, Austria	A ready-to-use surgical patch composed of a dry collagen sponge made from horse tendons, and on one side coated with human fibrinogen and thrombin	An adjunct to hemostasis in cardiovascular surgery when control of bleeding by standard surgical techniques (such as suture, ligature, or cautery) is ineffective or impractical.

Recothrom	2008	Zymogenetics, Seattle, WA	Recombinant thrombin	Aid in hemostasis whenever oozing blood and minor bleeding from capillaries and small venules is accessible and control of bleeding by standard surgical techniques is ineffective or impractical.

Evicel	2007 (Changed from Crosseal which was approved in 2003 when indication expanded to include use during vascular surgery)	Johnson and Johnson, Somerville, NJ; OMRIX biopharmaceuticals Ltd. Kiryat Ono, Israel	Fibrin sealant—human pooled	An adjunct to hemostasis for use in patients undergoing surgery (liver and vascular surgery are also separately indicated) when control of bleeding by conventional surgical techniques is ineffective or impractical.

Evithrom	2007	Johnson and Johnson, Somerville, NJ	Lyophilized human pooled thrombin	Aid in hemostasis whenever oozing blood and minor bleeding from capillaries and small venules is accessible and control of bleeding by standard surgical techniques is ineffective or impractical.

Thrombin-JMI	2007	King Pharmaceuticals, Bristol, TN	Bovine thrombin	Aid in hemostasis whenever oozing blood or minor bleeding from capillaries and small venules is accessible and control of bleeding by standard surgical techniques is ineffective or impractical.

CryoSeal Fibrin Sealant System	2007	Thermogenesis, Rancho Cordova, CA	Fibrin sealant-human	An adjunct to hemostasis on the incised liver surface in patients undergoing liver resection when control of bleeding by standard surgical techniques is ineffective or impractical.

Arista AH	2006	Medafor, Minneapolis, MN	Polysaccharide spheres	For use in surgical procedures (except neurological and ophthalmological) as an adjunctive hemostatic device to assist when control of capillary, venous, and arteriolar bleeding by pressure, ligature, and other conventional procedures is ineffective or impractical.

Vitagel	2006	Orthovita, Malvern, PA	Fibrin sealant-individual units of plasma, bovine collagen, and bovine thrombin	For use during surgical procedures (except neurosurgery and opthalmic surgery) as an adjunct to hemostasis when control of bleeding by ligature or other conventional procedures is impractical or ineffective.

CoStasis	2000	Cohesion Technologies Inc., Palo Alto, CA	Flowable bovine collagen and licensed bovine thrombin	In surgical procedures (other than neurological, opthalmological, and urological) as an adjunct to hemostasis, when control of bleeding by ligature or conventional procedures are ineffective or impractical.

TISSEEL	1998, new formulation in 2006	Baxter Healthcare Corporation, Westlake Village, CA	Fibrin sealant-human pooled	An adjunct to hemostasis in surgeries involving cardiopulmonary bypass and treatment of splenic injuries. TISSEEL is satisfactory for use in fully heparinized patients undergoing cardiopulmonary bypass. Also, indicated as an adjunct to prevent leakage from colonic anastomosis following the reversal of temporary colostomies.

Hemostase MPH	2006	Cryolife, Kennesaw, GA	Absorbable powder hemostatic	In surgical procedures (except neurological and opthalmological) as an adjunct hemostatic device when control of capillary, venous, and arteriolar bleeding by conventional means proves ineffective or impractical.

Surgiflo	2005	Johnson and Johnson, Somerville, NJ	Porcine gelatin with or without thrombin	In surgical procedures (except opthalmological) for hemostasis, when control of capillary, venous and arteriolar bleeding by pressure, ligature, and other conventional procedures is ineffective or impractical.

Duraseal	2005	Covidien, Waltham, MA	Single polyethylene glycol	An adjunct to sutured dural repair during cranial surgery to provide watertight closure. In 2011, additional approval for spine.

CoSeal	2003	Baxter Healthcare Corporation, Hayward, CA	Two polyethylene glycols	For use in vascular reconstructions to achieve adjunctive hemostasis by mechanically sealing areas of leakage.

Bioglue	2001	Cryolife, Kennesaw, GA	Bovine albumin and 10% glutaraldehyde	Adjunct to standard methods of achieving hemostasis (such as sutures or staples) in adult patients in open surgical repair of large blood vessels such as aorta or the femoral and carotid arteries.

Avitene Ultrafoam sponge and flour	2001	Bard, Murray Hill, NJ	Collagen-based absorbable hemostatic	For all surgical procedures including neurosurgery and urology as an adjunct to hemostasis when control of bleeding by ligature or conventional procedures is ineffective or impractical.

FloSeal Hemostatic Matrix	1999	Baxter Healthcare Corporation, Hayward, CA	Flowable bovine gelatin matrix and licensed human thrombin	In surgical procedures (other than ophthalmic) as an adjunct to hemostasis when control of bleeding by ligature or conventional procedures is ineffective or impractical.

Surgifoam sponge and powder	1999	Johnson and Johnson, Somerville, NJ	Porcine gelatin sponge	In surgical procedures (other than neurological, urological, and opthalmological surgery) as an adjunct to hemostasis when control of capillary, venous, and arteriolar bleeding by pressure, ligature, and other conventional procedures is ineffective or impractical.

Hemopad Novacol	1986	Datascope Corp., Montvale, NJ	Bovine collagen	As a hemostatic device, when control of capillary, venous, and arteriolar bleeding by pressure, ligature, and other conventional procedures is either ineffective or impractical.

Helistat Helitene	1985	Integra Life Science, Plainsboro, NJ	Bovine collagen	In surgical procedures (other than opthalmological and urological surgery) as an adjunct to hemostasis when control of bleeding by standard surgical procedure is impractical.

Instat, Instat MCH	1985	Johnson and Johnson, Somerville, NJ	Purified and lyophilized bovine dermal collagen	In surgical procedures (other than urological and ophthalmological surgery) as an adjunct to hemostasis when control of bleeding by ligature or conventional procedures is ineffective or impractical.

Gelfoam sponge and powder	1983	Pharmacia, Kalamazoo, MI	Porcine gelatin molded into a sponge	An aid in hemostasis, when control of capillary, venous, and arteriolar bleeding by pressure, ligature, and other conventional procedures is either ineffective or impractical.

CollaStat	1981	Integra Life Sciences Corporation, Plainsboro, NJ	Absorable bovine collagen sponge	In surgical procedures (other than opthalmological and urological surgery) as an adjunct to hemostasis when control of bleeding by standard surgical procedure is impractical.

Surgicel, SurgiCel Fibrillar, and Nu-Knit	1960	Johnson and Johnson, Somerville, NJ	Sponge of oxidized cellulose	Adjunct in surgical procedures to assist in control of capillary, venous, and small arterial hemorrhage when standard surgical techniques are ineffective or impractical.
